# Present status of renal replacement therapy in lower-middle-income Asian countries: Cambodia, Myanmar, Laos, Vietnam, Mongolia, and Bhutan as of June 2019 (before COVID-19), from the interviews of leading doctors in every country: (duplicated English publication from “the special Japanese edition of educational lectures in the 64th annual meeting of the Japanese Society for Dialysis Therapy”)

**DOI:** 10.1186/s41100-022-00443-2

**Published:** 2022-10-18

**Authors:** Toru Hyodo, Akihiro C. Yamashita, Nobuhito Hirawa, Yoshitaka Isaka, Hidetomo Nakamoto, Takashi Shigematsu

**Affiliations:** 1 The Committee of International Communication for Academic Research of the Japanese Society for Dialysis Therapy, Tokyo, Japan; 2 The Human Resource Development Program Committee for Dialysis Specialists in Developing Countries of the Japanese Society for Dialysis Therapy, Tokyo, Japan; 3 The Japanese Society for Dialysis Therapy, Tokyo, Japan; 4 The Congress Committee of the 64th Annual Japanese Society for Dialysis Therapy Meeting, Yokohama, Japan

## Abstract

The present status of Cambodia, Myanmar, Laos, Vietnam, Mongolia, and Bhutan as of June 2019 was reviewed from the interviews of leading doctors in every country. The timing was until just 6 months before the COVID-19 pandemic broke out. The cost per hemodialysis session was 25–70 US dollar and expensive if it is compared with average monthly income of every country. In Cambodia and Laos, patients must cover 100% of expenses for maintenance hemodialysis, in Myanmar, the government covers the cost of once-weekly dialysis sessions, in Vietnam, the government covers 80% of the cost, and in Mongolia and Bhutan, the cost is fully covered by the government. Continuous ambulatory peritoneal dialysis was widely available in Vietnam and Mongolia, which have achieved a relative standard of social infrastructure, but is far from common in any of the other countries. The number of patients on dialysis is increasing with economic development in all countries. Diabetic nephropathy is a common primary reason for dialysis. None of the countries discussed in this article had clinical engineers who could maintain hemodialysis equipment and carry out clinical tasks in dialysis centers. Hospitals were not maintaining their equipment, and damaged units were kept in storage to be used for spare parts. None of the countries had dieticians to provide patients with dietary guidance. Establishment and training of both clinical engineers and registered dietitians are major projects that must be undertaken.

## Introduction

Since 2015, the Committee of International Communication for Academic Research (CICAR) of the Japanese Society for Dialysis Therapy (JSDT) has held a symposium at the Society’s annual meeting that invites presenters from developing countries across Asia to help attendees better understand the current state of dialysis care in Asia and challenges in the field. JSDT has also established a travel grant program to incentivize more presenters to come to Japan and present abstracts at the meeting, giving them opportunities to introduce their academic work. Through these efforts, JSDT has forged close relationships with dialysis-related academic societies in these countries. JSDT has also further developed these relationships by establishing the Human Resource Development Program Committee (HRDPC) for Dialysis Specialist in Developing Countries of JSDT (chair: Akihiro C. Yamashita; vice chair: author of this paper, Toru Hyodo) with the aim of creating a program whereby young professionals working in dialysis from all these countries are invited to Japan for short training sessions. A pilot version of this program was launched in 2017 after volunteers with extensive knowledge of Asia were recruited from among members of the board of councilors. Many of these volunteers were also members of the non-governmental organization (NGO) Ubiquitous Blood Purification International (UBPI) who had actually visited these countries several times and had experience assisting local staff with dialysis. The Committee is currently developing a system to ensure that JSDT members across Japan can easily host young dialysis professionals from developing countries in Japan for training purposes, with the goal of transitioning from the pilot version to a full-scale version of the program around 2022.

As a result of these activities, CICAR and HRDPC have already built relationships that allow for free exchange of information with several Asian countries via email and other messaging services. Takashi Shigematsu, the chair of the 64th Annual Meeting of JSDT held in 2019, included a section on the status of dialysis care in developing Asian countries in that year as part of its educational lectures. The author gave a presentation as a representative of CICAR. The content was originally published in Japanese [1], and it was decided to republish it in English in *Renal Replacement Therapy*, the English-language journal of JSDT, as the information will be widely useful to readers around the world. This English duplication is accepted by the chair of the 64th Annual Meeting of JSDT, JSDT, and Tokyo Igakusha Co., Ltd..

## NGO ubiquitous blood purification international (NGO UBPI)

NGO UBPI is a non-governmental organization composed of member groups that was set up with an eye toward international development in the field of blood purification therapy. These member groups include JSDT, the Japanese Society for Technology of Blood Purification (JSTB), the Japan Association for Clinical Engineers (JACE), the Japanese Society for Artificial Organs (JSAO), and the Japanese Society of Renal Nutrition and Metabolism (JSRNM). The consortium aims to improve dialysis infrastructure in developing countries through initiatives in academic and technical education. NGO UBPI is an academic and professional organization composed of professionals such as clinical engineers, university professors, registered dieticians, nurses, physical therapists, and physicians.

## Present status of renal replacement therapy in lower-middle-income Asian countries as of 2019

### Cambodia

Cambodia has a population of about 16.5 million and a monthly substantial income of United States dollar (USD) 137 (1643 per year) as of 2019 [2]. Table 1 provides an overview of the status of dialysis in Cambodia in that year.

**Table 1:** Overview of dialysis in Cambodia (2018–2019)

Health insurance coverage for dialysis:	Patients bear 100% of the costs (maintenance dialysis)
Cost per HD session:	45–60 USD
Times of dialyzer reuse:	6
Length of dialysis session:	4 h
Number of dialysis sessions per week:	1–3 (depending on the patient’s financial situation)
Number of HD patients:	500–600 (in 2018)
Number of CAPD patients:	0 (not performed in 2019)

**Fig. 1:**
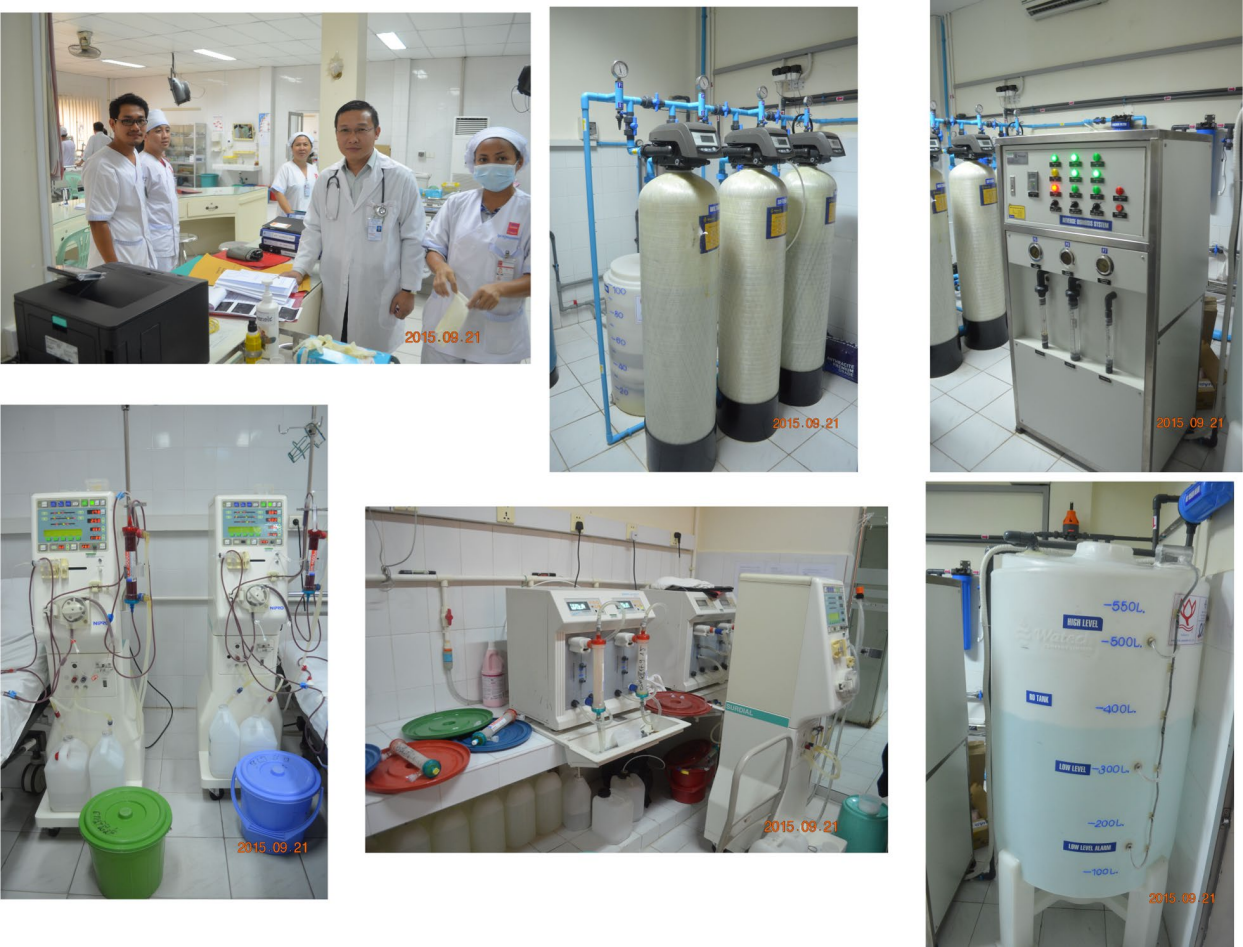
The dialysis center at Calmette Hospital equipped with dialysis machines similar to those used in Japan and other developed countries

Cambodia has launched a health insurance system, although as yet it is still inadequate. Patients must cover 100% of expenses for maintenance HD out of pocket, and each session costs USD 45–60. Dialyzers are reused 6 times, a dialysis session lasts 4 h, and dialysis is performed 1 to 3 times per week. There are 500–600 patients on hemodialysis. CAPD is not yet available, mainly because international manufacturers of peritoneal dialysis equipment have deferred entering the market due to the small patient population and the country’s underdeveloped distribution infrastructure. In early 2018, the Cambodian government began requiring businesses registered in Cambodia to enroll their employees in social insurance. Monthly premiums range from USD 3 to a maximum of USD 10.20. Public officials and members of the military get the maximum premium covered by the government and can receive the best medical care. Patients seeking treatment for an acute condition can access a better tier of medical care if they are paying higher premiums. It is particularly noteworthy that patients can now undergo acute hemodialysis for acute kidney injury. Patients can undergo 1 or multiple sessions depending on the premium they pay. Figure 1 shows the dialysis center at Calmette Hospital (the largest in Cambodia) equipped with dialysis machines similar to those used in Japan and other developed countries.

According to statistical data from the Cambodian Association of Nephrology (CAN), the number of patients on dialysis in Phnom Penh increased from 407 in 2017 to 499 in 2018 [3]. The total number of patients on dialysis is estimated to be about 100 more than this. Specifically, there are about 50 patients at a hospital in Siem Reap and about another 50 at a hospital in Battambang. These are the only 3 cities in the country that have dialysis centers. If residents from rural areas were to come to Phnom Penh for dialysis, they might have to travel 200–300 km and stay overnight in the city. Cambodia does not have public transit such as buses or trains, so patients must arrange their own transportation by, for example, motorcycle or car. If Japan had had no public transit system back in the time before dialysis became nearly free with social security, then a similar situation would have occurred, with only Tokyo, Osaka, and Nagoya having dialysis centers. The average monthly income in Cambodia is USD 137 only, so patients would have to sell their farm or home to come to the city for dialysis. As such, many patients with end-stage renal disease (ESRD) are forced to choose conservative therapy.

The proportion of male and female patients on dialysis is about 50/50. Median age is 55 years, and most patients are aged 51–60 years. Kidney biopsies are not performed in Cambodia, but the primary reason for dialysis is often diabetes, hypertension, or both. The average number of dialysis sessions per month is around 7–8 (7.8 ± 2.4 in 2017 and 7.2 ± 2.5 in 2018 [mean ± SD]) because patients pay for maintenance hemodialysis out of pocket [3].

CAN held its first annual meeting in November 2016 with the cooperation of NGO UBPI, and it has held meetings every year since. Besides organizing presentations related to kidney disease and dialysis, the association also organizes presentations on clinical engineering to communicate the importance of clinical engineers to local medical professionals and provide opportunities for Japanese clinical engineers to learn how to give educational presentations in English in foreign countries. This effort highlighted the urgent need for clinical engineers in Cambodia and led to the establishment of the Cambodian Association of Clinical Engineering. CAN became an International Society of Nephrology (ISN)-Affiliated Society in 2017, and elected a representative to the Oceania and South East Asia Regional Board at the 2019 World Congress of Nephrology in Melbourne. They had 2 abstracts accepted to the Congress and presented there. They also keep pace with ISN in holding a World Kidney Day event every year in March. The association also has a website (https://cambodia-nephrology.org/mainactivities.html) and publishes an official journal, *The Cambodian Journal of Nephrology* (ISSN 2518–0381) (with the support of Reiseikai Media).

### Myanmar

Myanmar is a large country in mainland Southeast Asia that is almost twice the size of Japan but with slightly less than half the population (53.6 million) and a monthly substantial income is about USD 119 (1422 per year) as of 2021 [4]. Table 2 provides an overview of dialysis care in Myanmar. Around 2016 to 2017, the government began fully covering the cost of once-weekly dialysis sessions at public hospitals. This is a major advancement considering that everyone other than the military had to pay completely out of pocket for dialysis care when I first visited Myanmar in 2013. However, due to the limited number of dialysis machines available, in reality patients often never get the chance to receive that once-weekly dialysis session to which they are entitled, forcing many poorer patients to choose conservative therapy and die. Patients who need dialysis more frequently than once a week can do so at a private hospital. A dialysis session costs USD 45 in total, but the government covers just USD10, so only those patients who can afford to pay the USD 35 balance are able to receive dialysis. CAPD costs USD 400, all of which must be paid for out of pocket. Dialyzers are reused up to 8 times, a dialysis session lasts 4 h, and the number of sessions per week depends on how much the patient can afford. For the educational lecture of the 64th annual meeting of JSDT, the author requested that the NIPRO Co. Ltd., medical equipment distributor in Myanmar conducted an interview survey with local physicians. They reported that 5% of patients were receiving dialysis 3 times a week, 70% twice a week, and 25% once a week. The estimated number of patients on hemodialysis is 4000. All the numbers presented so far have been rough estimates, but the number of patients on CAPD is exactly 66 (as of June 2019). No statistical surveys on renal failure have been conducted in Myanmar to date. The numbers presented should be considered rough estimates reflecting the status of dialysis care in 2019.

**Table 2:** Overview of dialysis in Myanmar (2018–2019)

No health insurance system available:	Publicly funded hospitals provide one free HD session per week for each patient. The government pays 10 USD and the patient pays 35 USD for an HD session at a privately funded hospital
Cost per HD session:	45 USD
CAPD costs:	400 USD per month
Times of dialyzer reuse:	Up to 8
Length of dialysis session:	4 h
Number of dialysis sessions per week:	1–3 times (depending on the patient’s financial situation)
	3: 200 patients (5%)
	2: 2800 patients (70%)
	1: 1000 patients (25%) (estimated figures)
Number of HD patients:	4000 (in 2019, estimated)
Number of CAPD patients:	66 (in 2019, by the direct information from CAPD doctors)

**Fig. 2:**
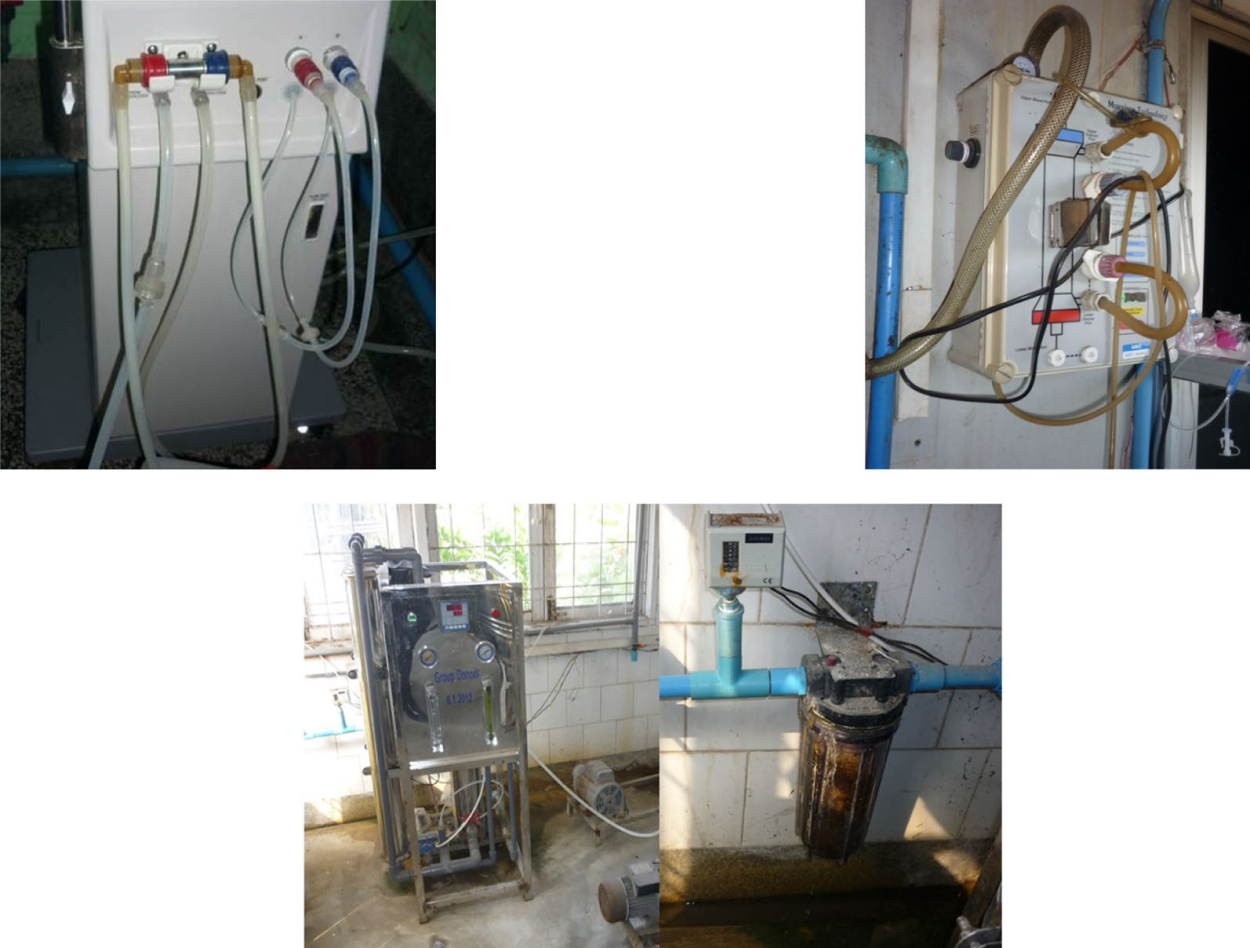
The dialysis machines were very dirty and in poor condition because they had been gifted by an international organization and the local hospital did not have staff capable of maintaining them

JACE, the Japanese Society for Hemodiafiltration (JSHDF), and NGO UBPI launched a joint project in Myanmar when Myanmar Yutani, the local NIPRO distributor, recommended presenters (Hideki Kawanishi, the President of JSHDF and NGO UBPI, Tomotaka Naramura, JACE, Masanori Shibata, JACE and the author, Toru Hyodo, the Secretary General of NGO UBPI) for the 1st Myanmar Nephro-Uro International Conference. When we visited Myanmar in 2013 to attend this conference, we toured a dialysis center and observed that the dialysis machines were very dirty (Fig. 2). The machines were in poor condition because they had been gifted by an international organization and the local hospital did not have staff capable of maintaining them. When we analyzed the dialysate and dialysis water for endotoxins and bacteria, we found that they were indeed highly contaminated and struggled to determine what part of the system—from the dialysate delivery system to the bedside console—would be the best place to start working on the problem. We concluded that with limited time and funds, the only option was to install an endotoxin retentive filter (ETRF). At the time, we were not confident as to whether an ETRF could effectively filter such highly contaminated dialysis water without clogging. Considering this a first step to go ahead and try, NGO UBPI sent a volunteer team of clinical engineers (including Tomotaka Naramura, Natsumi Abe, and Moe Kojima) to install ETRFs, and they were able to reduce the levels of endotoxins and bacteria in the dialysate to below the detection limit. No bacteria were detected in the dialysate when tested on-site 6 months and 1 year after ETRF installation.

**Fig. 3:**
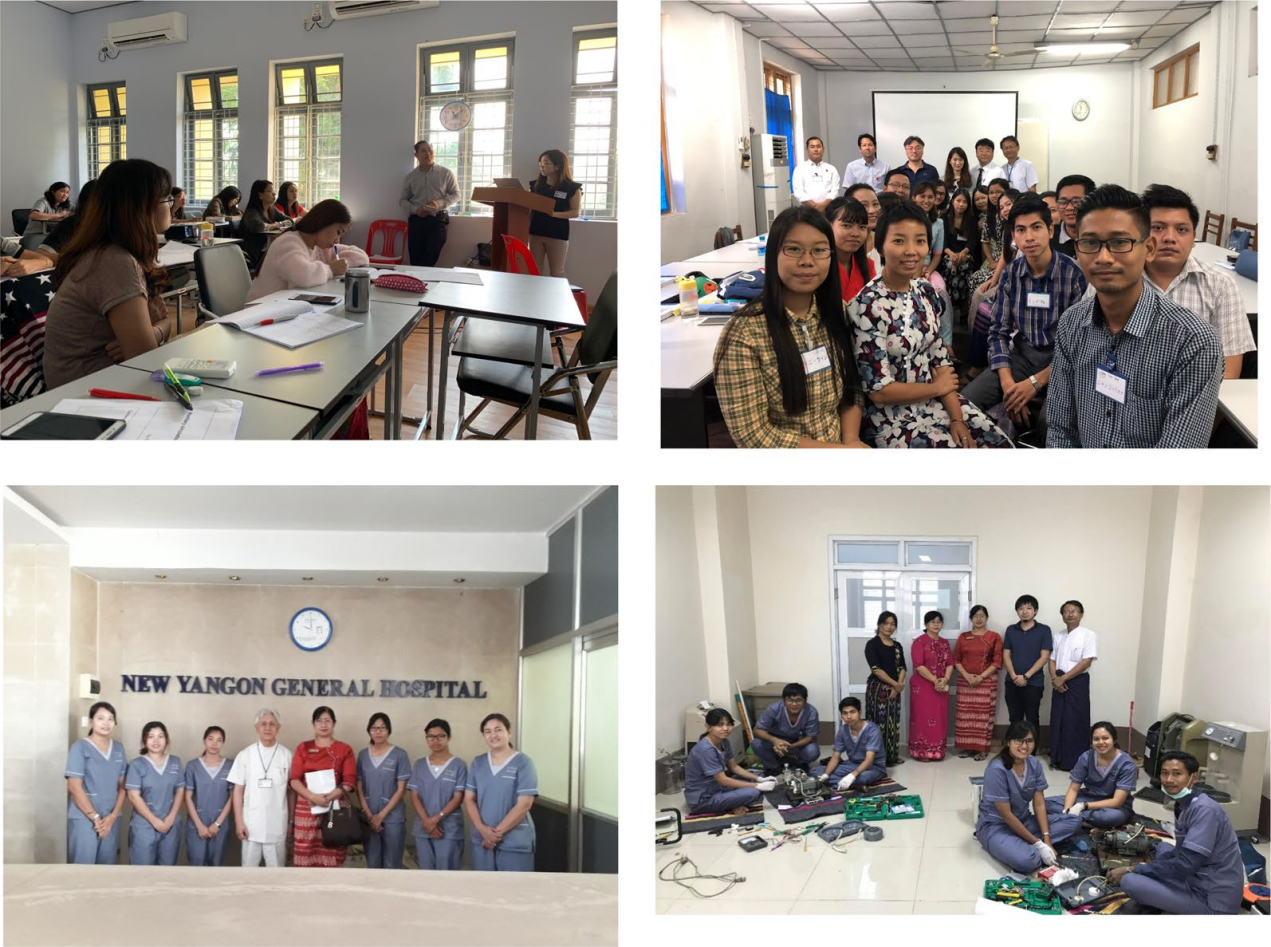
The scene of a lecture at the University of Medical Technology. Many clinical engineers from Japan took several days off from their regular work to participate in this JICA project on a rotating basis. The practical training sessions were practiced at Yangon General Hospital

In 2014, the Committee on International Affairs of JSTB (including committee chair Kenichi Kokubo, Shinichiro Urabe, and Teruhiko Miyamoto, etc.) joined the project to further spread the practice of dialysate purification by installing ETRFs in dialysis consoles across Myanmar. The project is now integrated with JSTB’s current initiatives to promote education of dialysis technicians in Myanmar. The data obtained from this project were published in an English-language journals as evidence that even developing countries need ETRFs in dialysis consoles [5–7]. The publication of these articles further advanced the move to install ETRFs in Myanmar, and both Japanese distributors in the country decided to make ETRFs standard in their dialysis consoles. Local physicians learned that these efforts were carried out not by doctors, but by clinical engineers who specialize in maintenance and care of medical equipment. Moreover, senior government officials in Myanmar also became aware that even if the country’s hospitals had the same machines and other such hardware as is available in Japan and Western countries, they still need to be staffed with skilled and knowledgeable clinical engineers. This prompted the launch of the Project for Human Resource Development of Medical Engineering, a venture mandated to the Clinical Engineering Global Promotion Foundation (CEGPF) (representatives: Tadayuki Kawasaki and Tomotaka Naramura) by the Japan International Cooperation Association (JICA). This is a large project started in 2018 with a budget of Japanese yen (JPY) 500 million and a 5-year timeline. The entrance ceremony for the first class of clinical engineers was held in June 2016, and a total of 36 clinical engineers have graduated in the 2 sessions completed as of April 2020. Students initially comprised radiologists, nurses, laboratory technicians, and engineering school graduates. Five students (2 in the first session and 3 in the second session) are currently participating in a 1-year program in Japan to be trained as faculty of the soon-to-be-established department of clinical engineering at the University of Medical Technology, Yangon. The other 16 students in the first session are working as clinical engineers in hospitals across Myanmar. The 15 students who graduated in April 2020 also plan to work at hospitals across the country. Many clinical engineers from Japan currently take several days off from their regular work to participate in this JICA project on a rotating basis (Motoko Kato, Shunichiro Urabe, Ako Hanaoka, Teruhiko Miyamoto, etc.). There are also a few permanent staff from Japan. Participating physicians include Toshihide Naganuma, Tunghuei Chang, and Toru Hyodo (the author). The practical training sessions were practiced at Yangon General Hospital (Fig. 3). The trainees are very enthusiastic and academically outstanding. They receive a salary from the government for the duration of the training period, but have to pay back a year of that salary if they fail the final exam to become a clinical engineer at the end of the program.

**Fig. 4:**
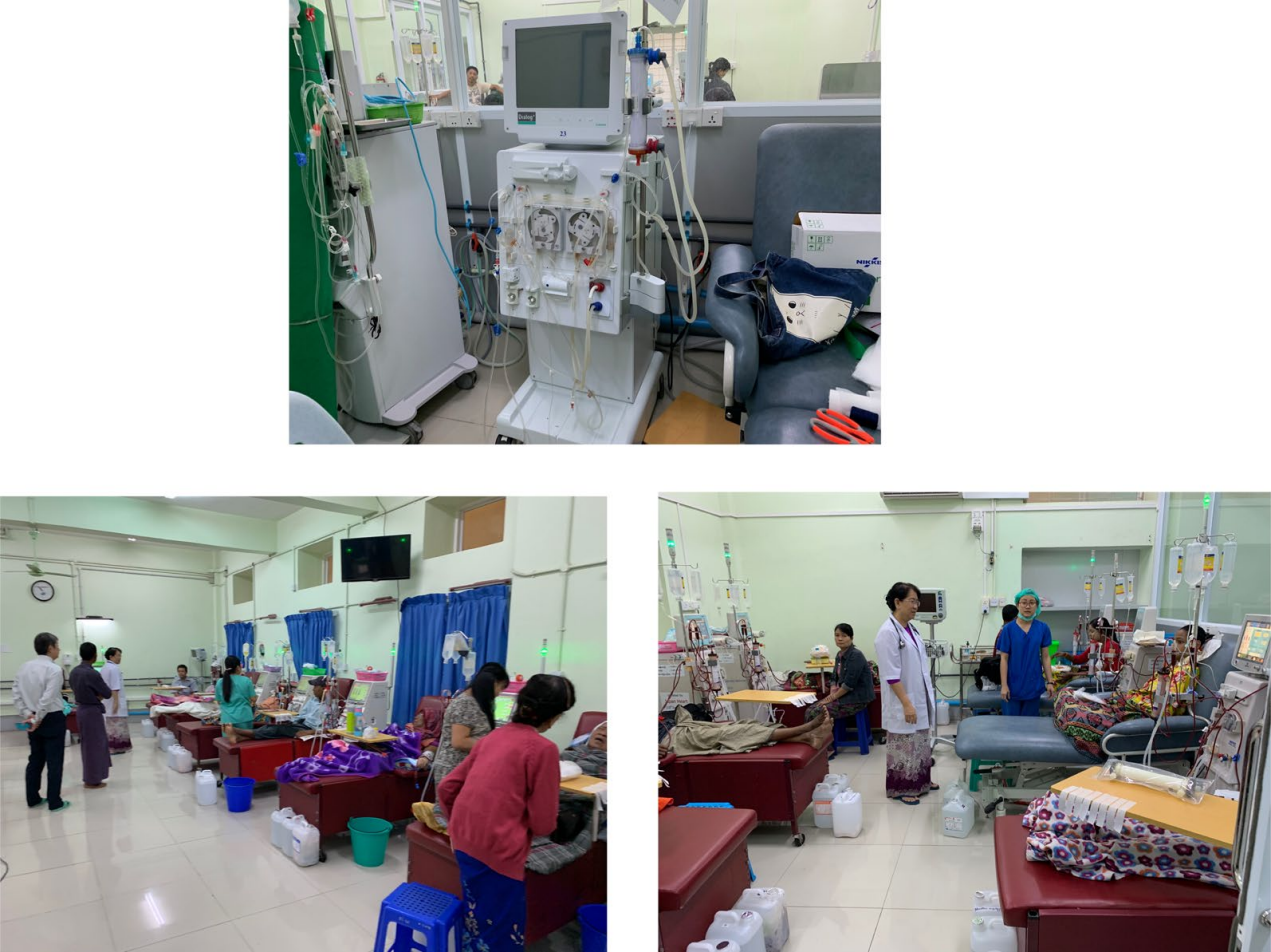
The Dialysis Center, Yangon Specialty Hospital, January 2019. The hospital is maintaining the same standards as Japan, even providing advanced care such as online hemodiafiltration and sustained low-efficiency dialysis for acute kidney injury

Lastly, the author would like to share what I saw when I visited Yangon Specialty Hospital (Director: Professor Khin Thida Thwin), Myanmar’s highest-level hospital, in 2019, 6 years after my first visit to the dialysis center of a public hospital in Myanmar (Sanpya Hospital, Center Director of the day: same as Prof. Khin Thida Thwin). The hospital is maintaining the same standards as Japan, even providing advanced care such as online hemodiafiltration and sustained low-efficiency dialysis for acute kidney injury (Fig. 4). This is a direct outcome of the free technical and knowledge support provided to the hospital by Tomotaka Naramura, Kenichi Kokubo, and other NGO UBPI members at the request of Professor Khin Thida Thwin. To raise other hospitals in Myanmar to the level of this hospital, Japanese organizations will continue to provide technical support for blood purification in Myanmar through various efforts, including inviting Japanese companies to install plumbing systems at low cost and the JICA project.

Information provided by:

Professor Khin Thida Thwin, Chair of the Myanmar Nephro-Urological Society, Yangon, Myanmar.

Mr. Yoshinori Komaru, Myanmar Yutani, Yangon, Myanmar.

### Laos

Laos, whose capital is Vientiane, is a country about two-thirds the size of Japan and a population one-seventeenth that of Japan, at 7.12 million. It is also a lower-middle income country, where citizens have an average monthly income of USD 223 (2670 per year) [8].

The Laotian health insurance system allows people to receive 6 free dialysis sessions in their lifetime, but all subsequent sessions must be paid for 100% out of pocket. A single dialysis session costs USD 50–70, dialyzers are reused 10–12 times, a dialysis session lasts 4 h, and the number of sessions per week ranges from 1 to 3 depending on how much the patient can afford. No statistical surveys on ESRD have been conducted in Laos, so the number of patients on dialysis is unknown (Table 3) [9]. In 2018, there were 3 patients on CAPD.

**Table 3:** Overview of dialysis in Laos (2016)

Health insurance coverage for dialysis:	During their lifetime, patients can receive 6 free dialysis sessions but subsequently bear 100% of the costs
Cost per HD session:	50–70 USD
Times of dialyzer reuse:	10–12
Length of dialysis session:	4 h
Number of dialysis sessions per week	1–3 (depending on the patient’s financial situation)
Number of HD patients:	Unknown
Number of CAPD patients:	0 (3 in 2018)

**Fig. 5:**
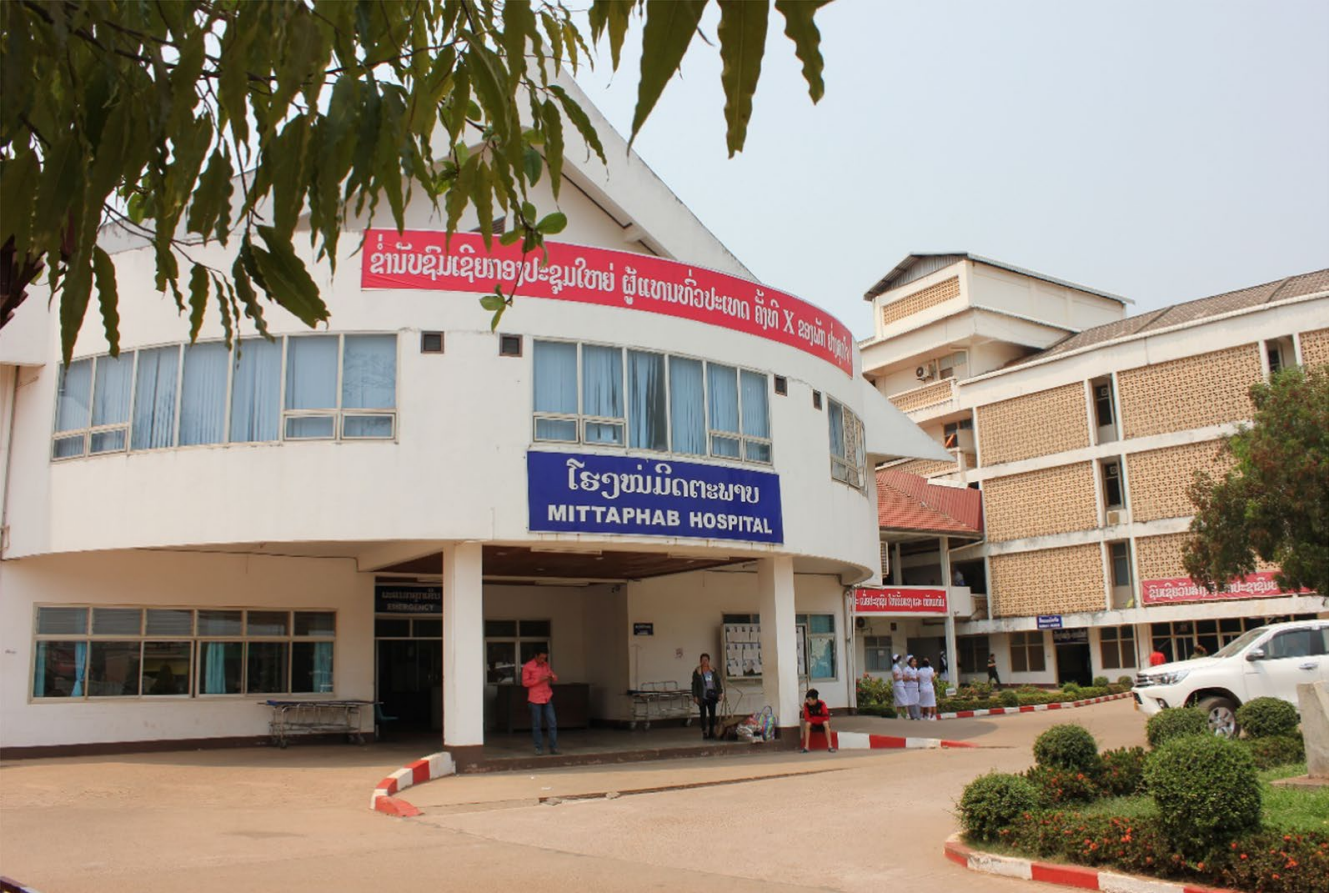
The Dialysis Center, Mittaphab Hospital, Vientiane, Laos: 2016

**Fig. 6:**
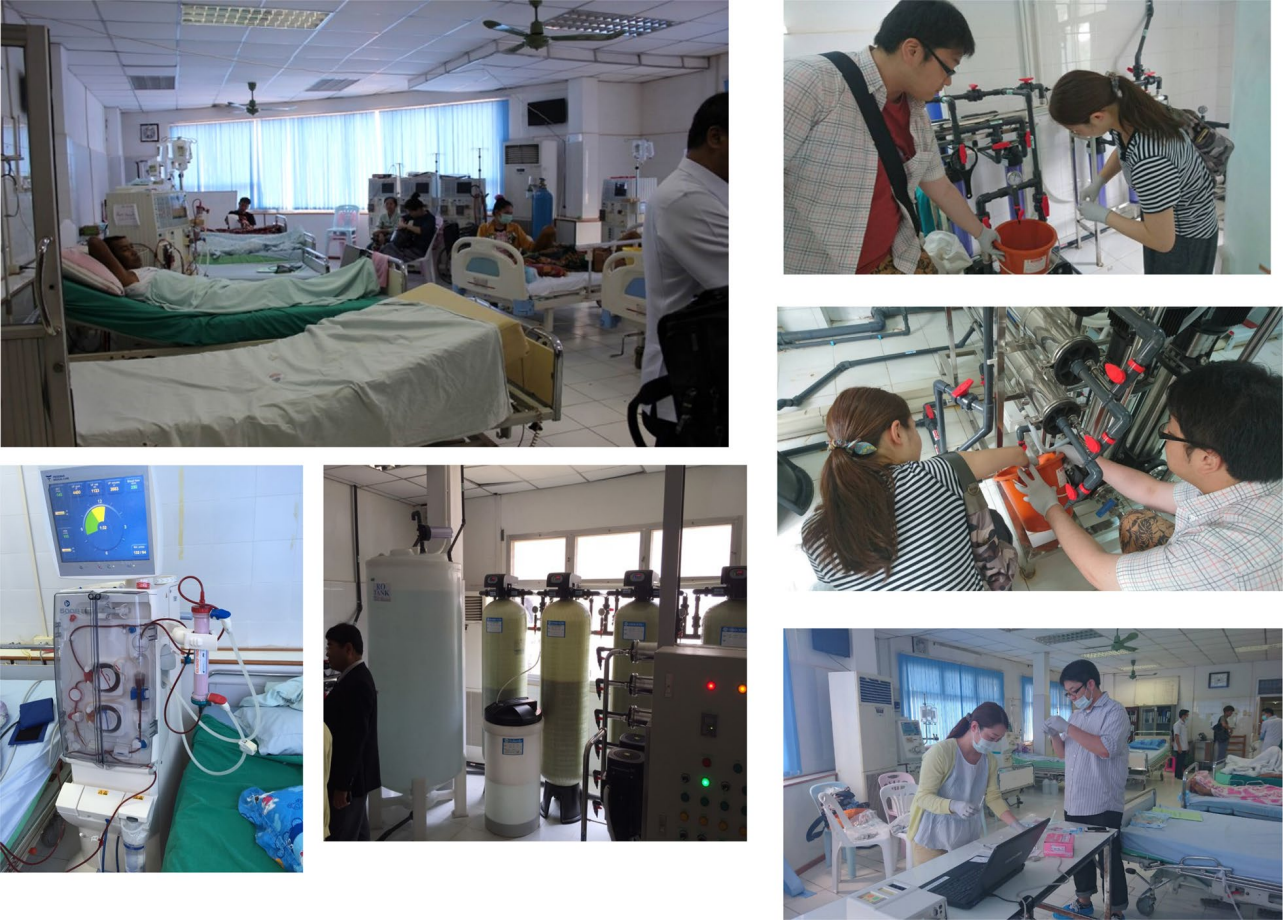
Two clinical engineers from NGO UPBI conducted water quality tests on the dialysis machines at the Dialysis Center, Mittaphab Hospital

Figure 5 shows Mittaphab Hospital, the flagship hospital for dialysis in Vientiane and the first hospital that NGO UBPI toured during our visit to the country in August 2016. The hospital had just received free dialysate delivery systems and dialysis consoles from the Japanese Tokushukai Medical Group the previous year, so the quality of the dialysis water was good. The donated dialysis consoles had ETRFs, and the levels of endotoxins and bacteria in the dialysate were both below the detection limit. However, the old dialysis consoles the hospital was using from before did not have ETRFs, and the purity of the dialysate from those consoles was clearly of worse quality than that from the new machines. Two clinical engineers from NGO UPBI, Takayuki Abe and Ayumi Takizawa, conducted water quality tests on the dialysis machines (Fig. 6). Local physicians observing their work asked questions about their profession and job duties. After learning of the role and duties of clinical engineers, the physicians realized that Laos needs them also. Considering that the hospital was equipped with machines used in Japan and other developed countries but that the staff were unable to maintain the machines, everyone agreed that clinical engineers needed to be trained in Laos as well.

**Fig. 7:**
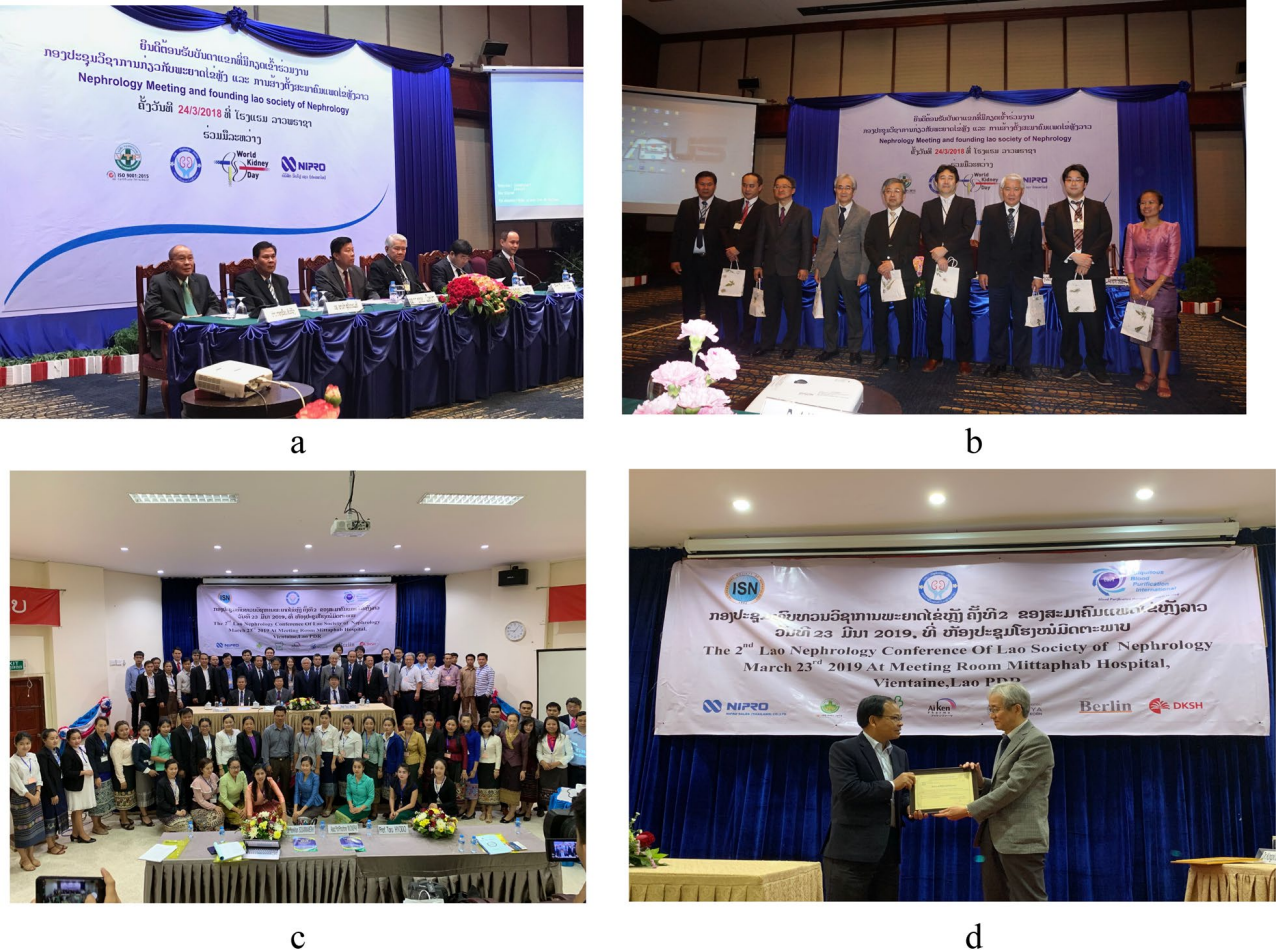
**a** and **b** are the first Annual Meeting of LSN. **c** and **d** are the second one. Dr. Kawanishi received the letter of thanks from LSN to the gift of money for Laos flood disaster from JSDT instead of the President of JSDT, Hidetomo Nakamoto (**d**)

The local physicians themselves would need to commit to improving their own practice before new staff specialized in dialysis-related machines could start being trained, so it was decided to establish a nephrology society in the country. The Lao Society of Nephrology (LSN) held its first conference in March 24, 2018. The conference was attended by the Vice Minister of Health of Laos, the Vice Chair of the Oceania and the South East Asia Regional Board of the International Society of Nephrology, Professor Kriang Tungsanga of Thailand, the President of the International Society of Blood Purification and NGO UBPI, Hideki Kawanishi of Japan, and NGO UBPI members Mizuya Fukasawa, Toshihide Naganuma, Takayuki Abe, Kenichi Kokubo, and Toru Hyodo. Over 100 Laotian physicians and nurses attended. Professor Phanekham Souvannamethy, director of the Kidney Center at Mittaphab Hospital, was appointed as the President of LSN (Fig. 7a, b). The second annual meeting of LSN was held in March 23, 2019 (Fig. 7c). Dr. Kawanishi received the letter of thanks from LSN and the government of Laos to the gift of money for Laos flood disaster from JSDT instead of the President of JSDT, Hidetomo Nakamoto (Fig. 7d).

Information provided by:

Professor Phanekham Souvannamethy, the president of LSN, Vientiane, Laos.

Dr. Noot Sengthavisouk, Mittaphab Hospital Kidney Center, Vientiane, Laos.

### Vietnam

Vietnam is of a similar size to Japan, with a land area of 330,000 km^2^, and a population of about 96 million compared with Japan’s 126 million. The monthly substantial income is USD 292 (3498 per year) as of 2020 [10].

**Fig. 8:**
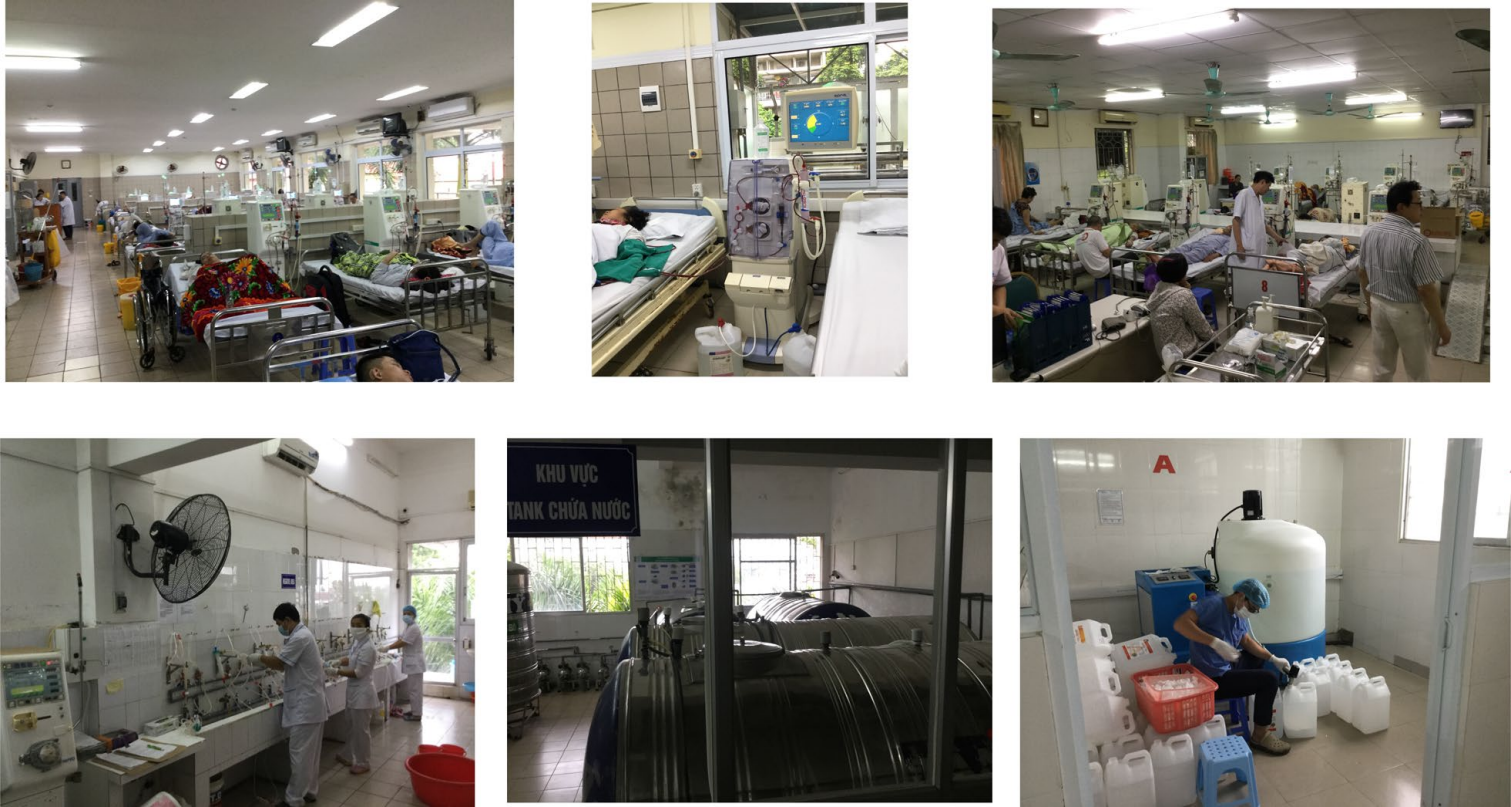
The dialysis center at Bach Mai Hospital, the largest hospital in North Vietnam. The center photo is a dialysis console for online HDF

Table 4 provides an overview of dialysis care in Vietnam. The government covers 80% of medical expenses. The total cost of a single dialysis session is the equivalent of 25 USD and the out-of-pocket cost to the patient is 5 USD. One month of hemodialysis costs 300 USD. CAPD is also available; 2 L is exchanged 4 times a day and 120 times a month, and a bag costs 3 USD. The total cost is 340 USD per month, and the out-of-pocket cost to the patient is 70 USD. A dialyzer is reused 6 times, each dialysis session lasts 3.5–4 h, and dialysis is performed 3 times per week. It is estimated that 22,000 patients are on hemodialysis and 2000 patients are on CAPD. The country has a health insurance system, but premiums vary depending on employment status and income. The system is set up so that people who pay higher premiums have access to better quality care, and patients pay about 30% of medical expenses out of pocket when they actually need care. Figure 8 shows the dialysis center at Bach Mai Hospital, the largest hospital in North Vietnam. The center photo is a dialysis console for online HDF. When NGO UBPI analyzed the quality of the dialysis water and dialysate, it met standards for online HDF set in Japanese guidelines.

**Table 4:** Overview of dialysis in Vietnam (2019)

Health insurance coverage for dialysis:	The government bears 80% of the costs
Cost per HD session:	25 USD (5 USD paid by the patient)
HD cost per month:	300 USD
CAPD	Four exchanges per day, 120 exchanges per month: one 2-L bag costs 3 USD
CAPD cost per month:	340 USD (70 USD paid by the patient)
Times of dialyzer reuse:	6
Length of dialysis session:	3.5–4.0 h
Number of dialysis sessions per week:	3
Number of HD patients:	22,000 (in 2019, estimated)
Number of CAPD patients:	2000 (in 2019, estimated)

**Fig. 9:**
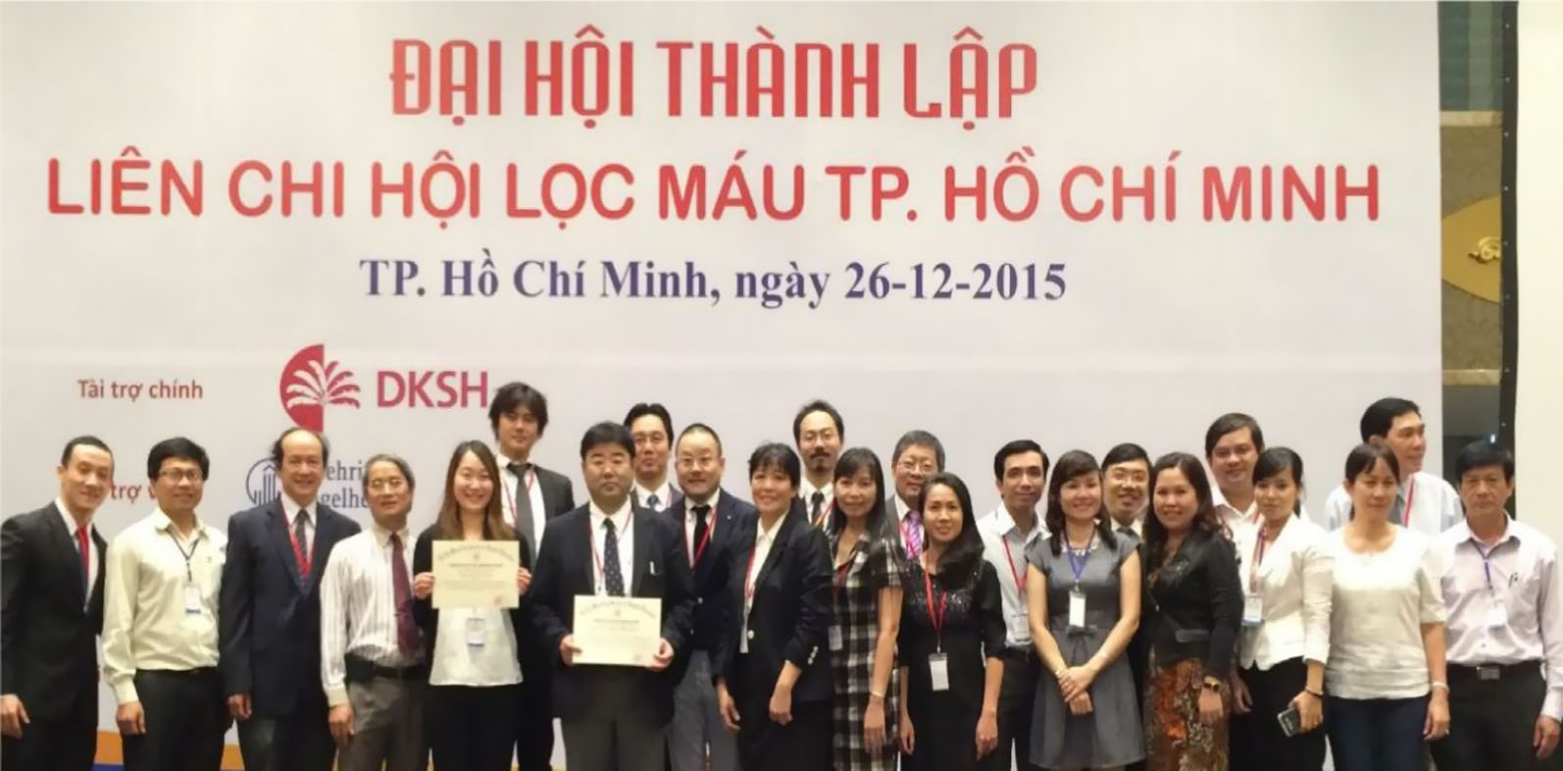
The first annual meeting of HSDT in 2015

**Fig. 10:**
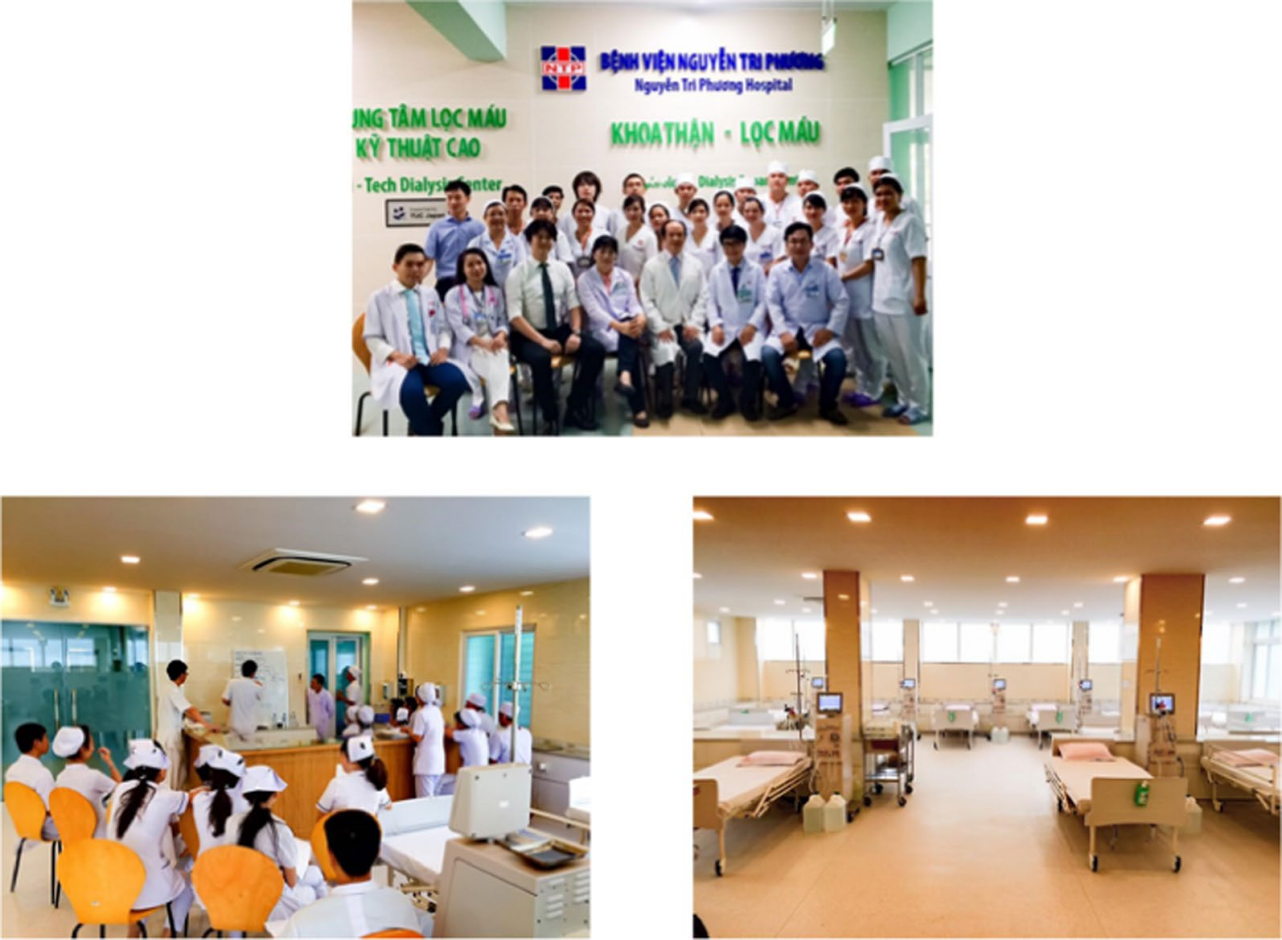
High Tech Dialysis Center, Nguyen Tri Phuong Hospital with Japanese management practices and standards

Vietnam did not originally have a society like JSDT that enables dialysis professionals to act collectively. A team led by Professor Pham Van Bui, who attends JSDT meetings every year, recognized the need for a similar society in Vietnam and set up the Ho Chi Minh City Society of Dialysis Therapy (HSDT) in 2015 (Fig. 9). HSDT held its first annual meeting that same year. Clinical engineer Ayumi Takizawa and engineering professor Kenichi Kokubo of NGO UBPI visited from Japan and emphasized the importance of clinical engineers. NGO UBPI member Dr. Atsushi Ueda and JSRNM President Satoko Tamura presented at the second annual meeting of the HSDT held in Da Nang in 2016. Presentations by NGO UBPI President Hideki Kawanishi and registered dietician Yukie Kitajima, who explained the process of CAPD and emphasized the importance of dieticians, were organized for the third annual meeting held in Ho Chi Minh City in 2017. In 2018, Hideki Kawanishi similarly presented on blood purification, and NGO UBPI Secretary General Toru Hyodo presented on the importance of carbohydrate counting in dietary therapy for diabetic patients on dialysis. A dialysis center with Japanese management practices and standards was established at Nguyen Tri Phuong Hospital (High Tech Dialysis Center, Director: Professor Pham Van Bui) cooperating with TUC Vietnam in 2016 (Fig. 10).

Information provided by:

Professor Pham Van Bui, President, HSDT, Ho Chi Minh City, Vietnam.

Dr. Bao Ngoc Nguyen, Department of Nephrology, Bach Mai Hospital, Hanoi, Vietnam.

Dr. Dinh Duc Long, Kidney Center, Bach Mai Hospital, Hanoi, Vietnam.

Mr. Nguyen Nam Duc, President, TUC, Vietnam, Ho Chi Minh City, Vietnam.

### Mongolia

Mongolia covers a large expanse of territory with a land area of 1,564,116 km^2^, roughly 4 times that of Japan, but has a much smaller population, at only 3.35 million. The monthly substantial income of USD 346 (4151 per year) as of 2020 [11].

Dialysis is covered 100% by health insurance, but patients must pay for medications out of pocket. Each dialysis session costs USD 50, and the monthly cost is USD 600. CAPD solution is exchanged 4 times per day, the monthly cost is USD 753, and domestically manufactured CAPD solution is available in Mongolia. Dialyzers are single use, each dialysis session lasts 4 h, and dialysis is performed 3 times per week. The number of patients on hemodialysis is 753, and the number on CAPD is 66 (Table 5).

**Table 5:** Overview of dialysis in Mongolia (as of December 31, 2018)

Health insurance coverage:	Dialysis costs are fully covered by health insurance
	Patients fully bear the costs of medicines
Cost per HD session:	50 USD, 600 USD per month
CAPD	Four exchanges of 2-L bags per day, costing 753 USD monthly
	Domestically produced CAPD fluids and preparation kit
Times of dialyzer reuse:	Single Use
Length of dialysis session:	4 h
Number of dialysis sessions per week:	3
Number of HD patients:	753 (in 2019)
Number of CAPD patients:	66 (in 2019)

**Fig. 11:**
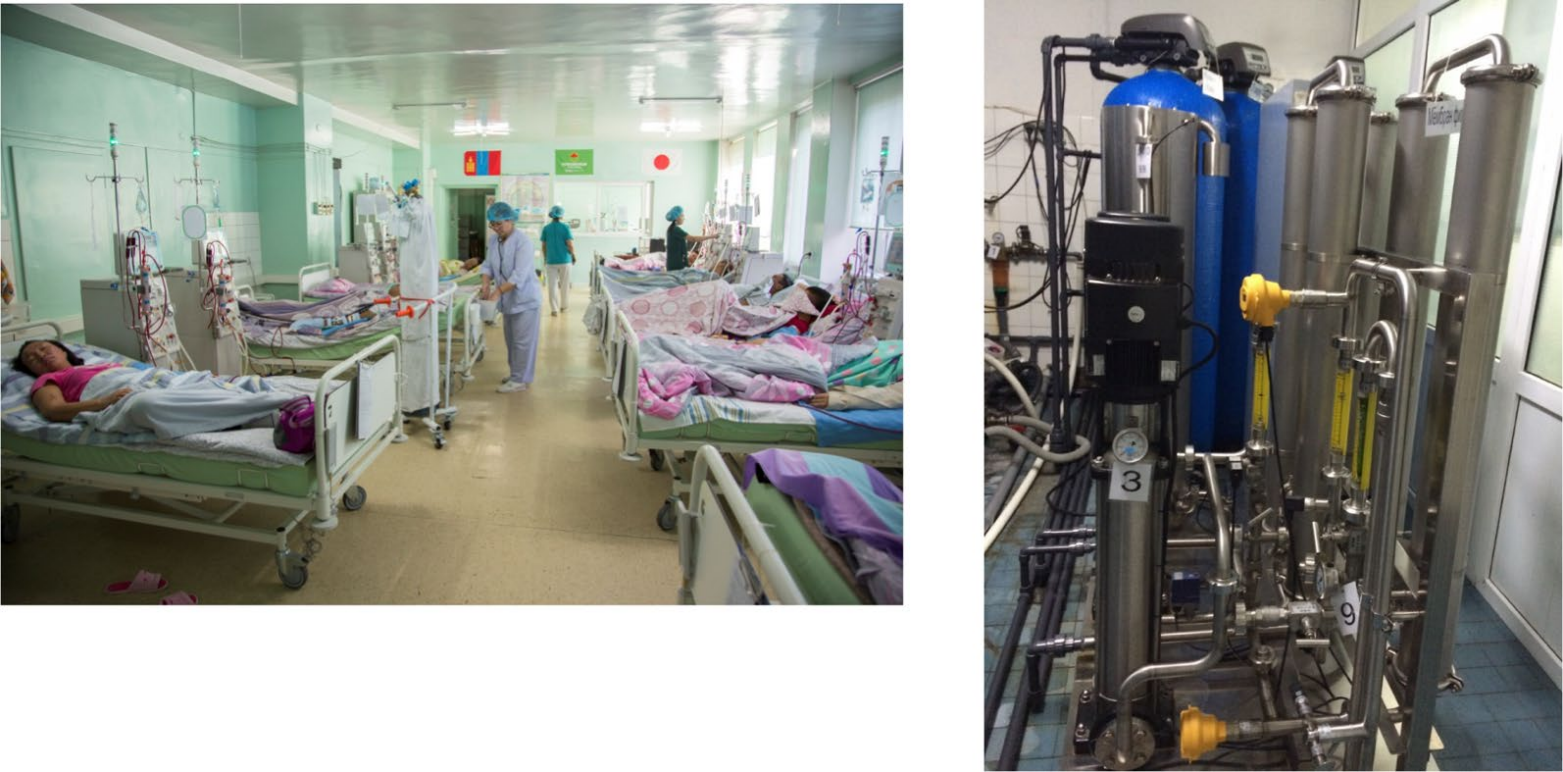
The scene of Dialysis Center at First Central Hospital of Mongolia. The storage tanks for dialysis water were a suitably small size for dialysate purification

**Fig. 12:**
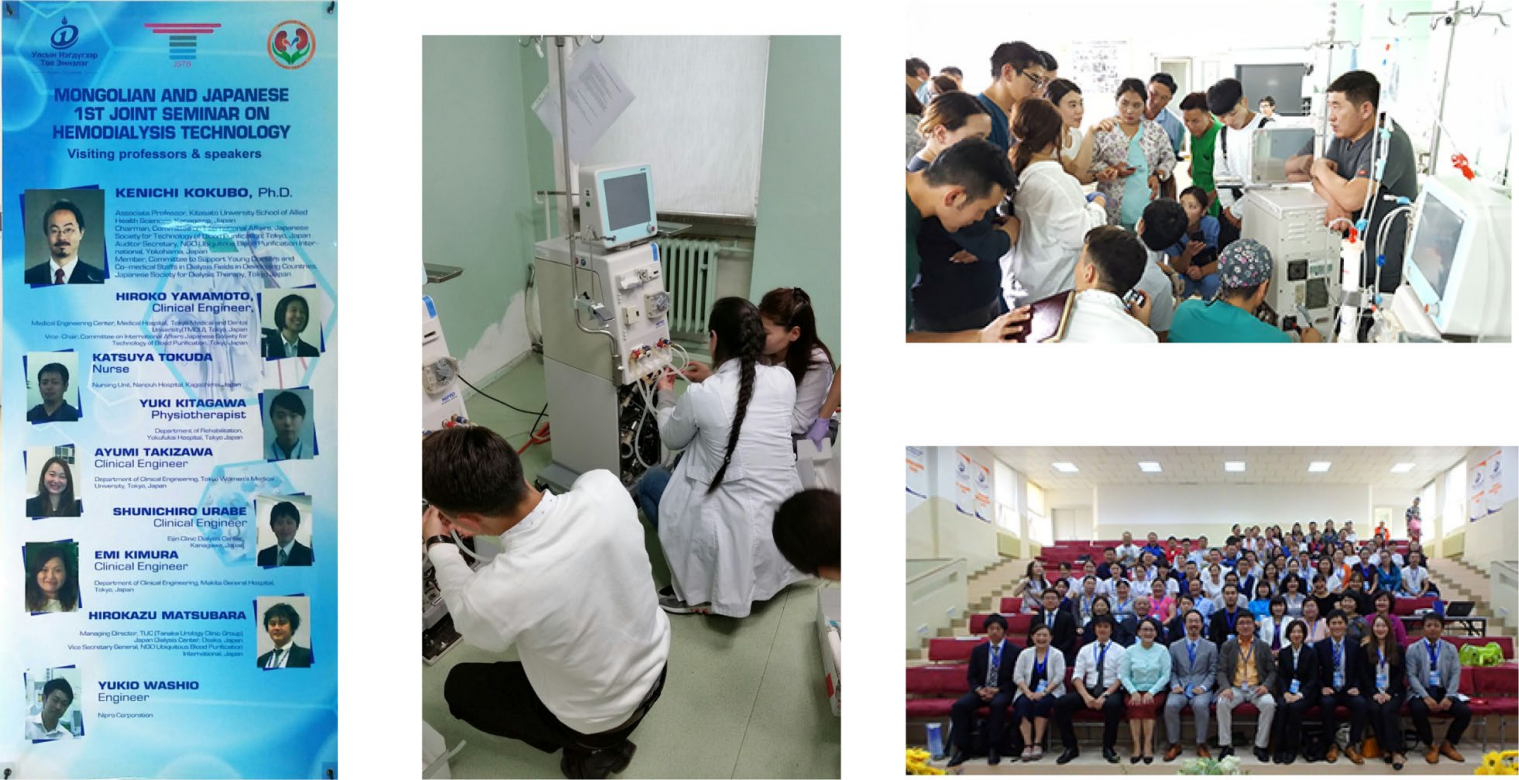
JSTB and NGO UBPI hold seminars at First Central Hospital of Mongolia each year. At hands-on seminars, presenters show attendees methods to test for bacteria and endotoxins

Figure 11 shows the dialysis center at First Central Hospital of Mongolia, which was established with the support of the Japanese Tokushukai Medical Group. The dialysate quality was good and, as would be expected from a dialysis center created with Japanese assistance, the storage tanks for dialysis water were a suitably small size for dialysate purification. The facility did not routinely test the water for endotoxins or bacteria, but results of the tests that were run were good. At the request of the Mongolian Society for Dialysis Therapy, JSTB and NGO UBPI hold seminars at this hospital each year. At hands-on seminars, presenters show attendees methods to test for bacteria and endotoxins (Fig. 12).

Information provided by:

Dr. Chuluuntsetseg Dorji, President of the Mongolian Society for Dialysis Therapy, Ulaanbaatar, Mongolia.

Dr. Saruultuvshin Adiya, Kidney Center, First Central Hospital of Mongolia, Ulaanbaatar, Mongolia.

### Bhutan

Bhutan is roughly one-tenth the size of Japan (38,394 km^2^) and has a much smaller population, at only 754,000. The monthly substantial income of USD 260 (3117 per year) as of 2018 [12].

**Fig. 13:**
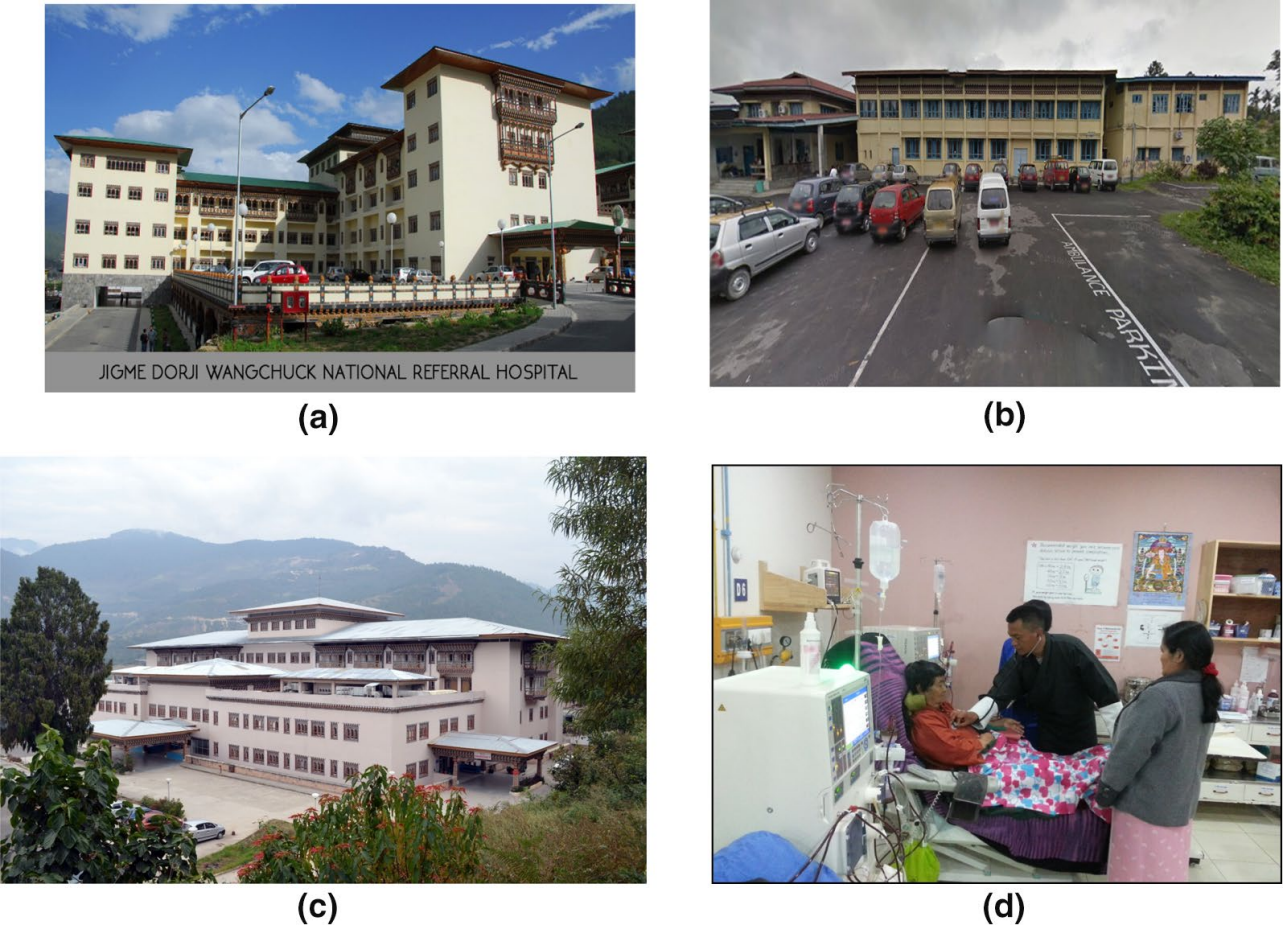
**a** National Referral Hospital, Thimphu, established a dialysis room at 1998. **b** Mongar Regional Referral Hospital has been equipped with it since 2011. **c** Gelephu Regional Referral Hospital also since 2011. **d** The scene at the dialysis room of National Referral Hospital, Thimphu

Medical expenses are covered 100% by the government. A dialysis session costs $30, dialyzers are reused 3 times, a dialysis session lasts 4 h, and dialysis is performed twice a week. The number of patients on hemodialysis was 150 in 2016 [9], and CAPD is not available in the country as of 2019 (Table 6). A physician from Bhutan planned to come to Japan to learn how to perform CAPD, as part of a program launched by HRDPC in February 2020 in vain due to COVID-19 pandemic. Bhutan has dialysis centers at 3 hospitals. There are no official statistics on patients with ESRD, but there are known to be about 150 patients on dialysis across the country. The 3 dialysis centers together have a total of 18 dialysis consoles (Fig. 13).

**Table 6:** Overview of dialysis in Bhutan (2016–2019)

Health insurance coverage:	The government bears 100% of medical costs
Cost per HD session:	30 USD
Times of dialyzer reuse:	3
Length of dialysis session:	4 h
Number of dialysis sessions per week:	2
Number of HD patients:	150 (in 2016)
Number of CAPD patients:	0(not performed in 2019)

The number of patients on dialysis increased 50-fold from 1998 to 2015. Most are working-age people aged 31–50 years, with a slight male preponderance (male: 54%, female: 46%). The distribution of primary reasons for dialysis is unknown because biopsies are not performed, but it appears that diabetes accounts for a surprisingly small percentage only (13%). From 1998 to 2015, 25% of patients on dialysis underwent kidney transplantation, and 75% continued hemodialysis.

Information provided by:

Dr. Minjur Dorji, Kidney Center, Jigme Dorji Wangchuck National Referral Hospital, Thimphu, Bhutan.

## Conclusions

Dialysis therapy is generally available in Cambodia, Myanmar, Laos, Vietnam, Mongolia, and Bhutan; however, the cost is still a heavy burden in these lower-middle income countries. Diabetic nephropathy is a common primary reason for dialysis. None of the countries had dieticians to provide patients with dietary guidance. And they had no clinical engineers who could maintain hemodialysis equipment and carry out clinical tasks in dialysis centers. Hospitals were not maintaining their equipment and damaged units were kept in storage to be used for spare parts.

Establishment and training of both clinical engineers and registered dietitians are major projects that must be undertaken.

## Data Availability

Not applicable.

## Abbreviations

HD: Hemodialysis
USD: United States dollar
CAPD: Continuous ambulatory peritoneal dialysis
CICAR: The Committee of International Communication for Academic Research
JSDT: The Japanese Society for Dialysis Therapy
HRDPC: The Human Resource Development Program Committee
NGO: The Non-Governmental Organization
UBPI: Ubiquitous Blood Purification International
JSTB: The Japanese Society for Technology of Blood Purification
JACE: The Japan Association for Clinical Engineers
JSAO: The Japanese Society for Artificial Organs
JSRNM: The Japanese Society of Renal Nutrition and Metabolism
CAN: The Cambodian Association of Nephrology
ESRD: End-stage renal disease
ISN: The International Society of Nephrology
JSHDF: The Japanese Society for Hemodiafiltration
ETRF: Endotoxin retentive filter
CEGPF: The Clinical Engineering Global Promotion Foundation
JICA: The Japan International Cooperation Association
JPY: Japanese yen
LSN: The Lao Society of Nephrology
HSDT: The Ho Chi Minh City Society of Dialysis Therapy
